# Need for Ethical Governance on the Implementation and Use of Patient-derived Xenograft (PDX) Models for Anti-cancer Drug Discovery and Development: Ethical Considerations and Policy Proposal

**DOI:** 10.31662/jmaj.2023-0199

**Published:** 2024-08-09

**Authors:** Kenji Matsui, Shigehiro Yagishita, Akinobu Hamada

**Affiliations:** 1 Division of Bioethics and Healthcare Law, National Cancer Center Institute for Cancer Control, Tokyo, Japan; 2 Division of Molecular Pharmacology, National Cancer Center Research Institute, Tokyo, Japan

**Keywords:** patient-derived xenograft model, research ethics, ethical governance policy, trust

## Abstract

Patient-derived xenograft (PDX) models, in which tumor tissues resected from cancer patients are transplanted into immunocompromised mice, have been recently considered the most reliable preclinical models that quite accurately stimulate the real-world characteristics and microenvironments of tumors in patients. The ethical uniqueness of the PDX model, which lies in the fact that it is a hybrid of living human tumor tissue and a carrier mouse, raises several ethical concerns. This study presents four ethical points for consideration and a model ethical governance policy for the implementation and use of PDX models for research. First, PDX models carrying living tumor tissues originating from individual patients with dignity must be treated ethically as materials and data in compliance with the principle of respect for persons. Second, although PDX models themselves are patentable and can be commercialized, it is a standard view, as represented by the Oviedo Convention by the Council of Europe, that those living tumor tissues carried by PDX models shall not give rise to financial gain since those tissues are human body parts; therefore, they should be treated according to a recent ethical approach with the *custodianship model* as the trust property of patients of origin and shall not be subjected directly to monetary transactions. Third, PDX models must be treated with due care in an ethical manner in line with experimental animal ethics. Finally, the implementation and use of PDX models for research purposes must comply with national and international regulations on both animal experimentation and human subject research. These four points should be carefully examined and properly institutionalized as an ethical governance policy in each institution that plans on utilizing or implementing PDX models for research.

## Introduction

**Figure 1.**
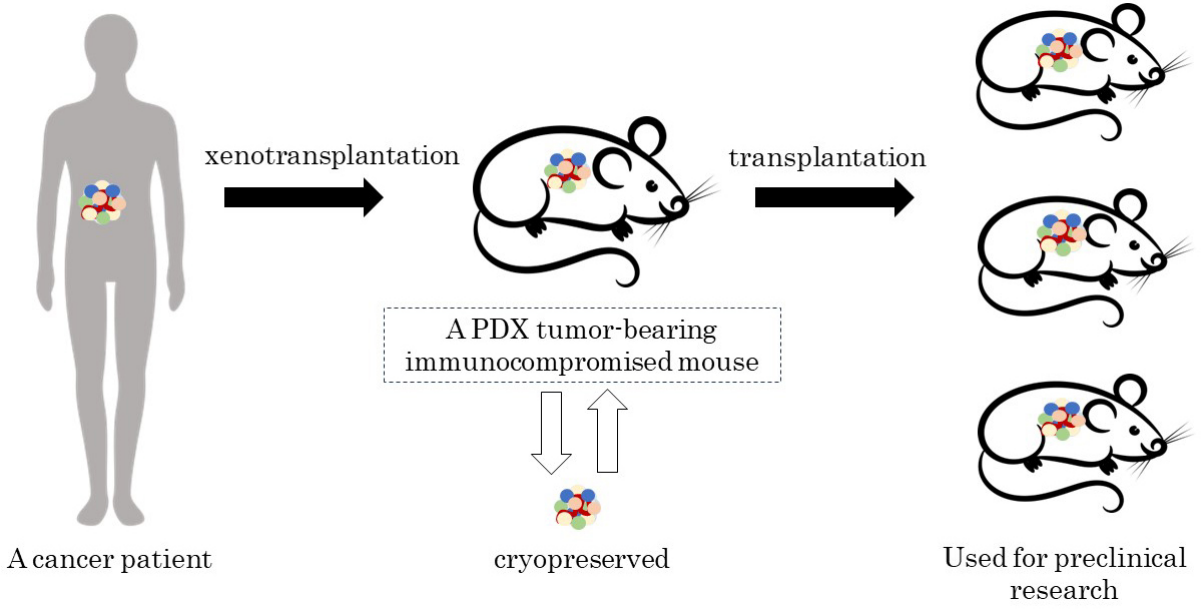
Schema of PDX model development.

The development of a novel drug requires an enormous amount of time and money. However, most chemical compounds, being found to be effective in preclinical studies and identified as new drug candidates, do not make it through clinical trials, with only about 5 to 10% being successfully approved for marketing ^(1)^. This is particularly true and noticeable in the development of anticancer drugs. Meanwhile, patient-derived xenograft (PDX) models, in which fresh tumor tissues resected from cancer patients are transplanted into immunocompromised mice (Figure 1), have come into the limelight as the most reliable preclinical models of genuine cancer status in real-world patients. Moreover, since PDX models reflect human intratumor heterogeneity and recapitulate the three-dimensional tumor architecture, they are currently considered one of the most promising models because they faithfully simulate the characteristics and microenvironments of tumors in patients of origin. PDX models have been used in identifying prognostic biomarkers, evaluating the efficacy of novel drugs, screening drug-sensitive and drug-resistant patients, and exploring drug-resistance mechanisms ^(2)^.

The US National Cancer Institute decided in 2016 to replace human cancer cell lines with PDX models for use in the screening of most drugs, with the establishment of a newly launched rejuvenated repository of PDX models ^(3)^. Similarly, European institutions launched a PDX consortium, called EurOPDX, in 2013, boasting a panel of more than 1,500 PDX models. In Japan, the National Cancer Center (NCC) established a large-scale domestic repository of PDX models in 2018, and currently, over 600 PDX models are available through this repository, referred to as the J-PDX Library.

Thousands of human-in-mice models are stored, or “biobanked,” in each of the major PDX repositories. Thus, scientists can easily access living human cancer tissues in the repositories whenever they want, along with detailed information about the patients who provided tissue samples with consent for use in the development and subsequent use of PDX models for research purposes. As such, those “secondary” researchers, or PDX users, may not be as aware of the ethical issues, including informed consent, as “primary” researchers (i.e., PDX developer/biobanker) may be. Moreover, from the perspective of PDX users, the material provided by a PDX biobank is perceptibly a mouse (or a lump of tissue taken from the mouse, sometimes), seemingly requiring no conscious thought that it is actually a hybrid partly originating from a human source. This moral uniqueness of biobanked PDX materials and the ease of acquisition for PDX users, however, may give rise to ethical problems, such as abusing human-originating tissues or selling them to a third party at their discretion; thus, it raises ethical concerns for the PDX bonafide biobanking researchers who have contracted with patients donating the tissues. Therefore, PDX biobanks must define a clear ethical policy, setting prudent rules for the implementation of the repository and the use and provision of their PDX models.

## Core Ethical Considerations on PDX Models

As previously noted, since a repository of PDX models has morally unique, hybrid characteristics of a biobank of both mice and human living tissues carried by the mice, we briefly present four ethical considerations that should be institutionally examined in establishing and/or utilizing a PDX biobank for research.

### Consideration 1: PDX as a source of living human materials involving dignity

PDX models are carriers of living tumor tissues that originate from individual patients who have dignity, whether dead or living. Although in some countries research on unidentifiable or deidentified human materials and clinical data may not be considered human subject research, it does not necessarily mean that scientists are unconditionally permitted to treat the materials and data as mere nonhuman things. Also, in countries including Japan, such research is still regarded as human subject research (i.e., even if materials and data are deidentified) and researchers are required to treat individual materials and data in an ethical manner based on the principle of respect for persons. This is one reason why research using a PDX model is usually required to be reviewed and approved by a human subject research ethics committee before implementation, in addition to a review by an animal care and use committee.

Since creating PDX models equates to developing expost human-animal chimera, strong resistance from the general public might be expected at present. Therefore, it is crucial to implement it according to the individual’s informed consent. However, when it is already assumed at the time of initial consent for a study or a biobanking project that the tumor tissues collected from their participants will be subsequently provided for developing PDX models, and only if that has been explained to those participants, then consent for their secondary use in developing PDX models can be waived, and using opt-out procedures will be permissible.

### Consideration 2: Trust-based ethical governance

A PDX model itself is patentable and can be commercialized. However, since tumor tissue carried by a PDX model is not only derived from a patient but is also associated with the patient’s personal information, it is considered human material. As the Oviedo Convention by the Council of Europe clearly provides, any human body parts “shall not give rise to financial gain,” and materials of human origin including tumors are widely perceived as matters beyond commercialization.

In the ethical discourse on human biobanks in general, this standard view of noncommercialization of human materials has long been controversial and inconclusive. In contrast, a new model of approach, referred to as the “custodianship model,” has recently attracted wide attention. Based on the model, “biobanks would undertake the role of the trusted intermediary and demonstrate accountability,” and “[c]ustodianship does not entail the right to ownership” and “does not regard a biospecimen as commodity for profit making” ^(4)^. It is important to note, however, that the custodianship model does not necessarily deny making profits from human materials. In fact, it does allow the beneficiary of the trust to receive the profits generated from the trust assets.

The custodianship model should be used as an ethical governance scheme at the time of implementation and use of PDX models in research. The patient-derived tumor tissue in a PDX model should remain the trust property of the patient of origin and not be subjected directly to monetary transactions, but both PDX biobanking researchers and PDX users should be allowed to gain reasonable profits, if any, generated from the utilization of the trust assets, namely, the tissues and the associated clinical information of the patient ^(5)^. Under the custodianship model, then, the PDX biobank is obliged to manage and control the tissues and the information appropriately and consistently with the consented purposes of the trust by the originated patients.

### Consideration 3: Gratitude and due care for human-in-mice models

The regulatory requirement that necessitates drugs in development to undergo testing in animal models before clinical trials for human participants is gradually being eased, a recent trend that partly reflects a practical approach and humanitarian principle under strong pressure by animal rights advocates. However, it is unavoidable for research with PDX models to use laboratory human-in-mice for preclinical experimentation. Researchers will sacrifice the lives of those mice instead of human patients for the benefit of human society and scientific developments, including the development of novel anticancer drugs. They are therefore well-deserved to be treated with due care in an ethical manner in compliance with the 3Rs―refinement, reduction, and replacement―which are currently understood as the international ethical principles for protecting the welfare of laboratory animals. We must also be profoundly grateful for their sublime sacrifice for the good of human society.

### Consideration 4: Regulatory compliance

Considering that the PDX models contain human-originated materials, including tissue samples and personal data of patients of origin, the implementation and use of PDX models for research purposes must follow regulations on both animal experimentation and human subject research. As for animal experimentation, Japan has established the Act on Welfare and Management of Animals (Act No. 105 of 1973), specifying the duty of due care with the 3Rs for laboratory animals; this must also be applied to the use of PDX models. In addition, international agreements such as the Cartagena Protocol and the Nagoya Protocol might be applicable for some research involving PDX models.

With regard to the human subject research, conversely, diverse regulations and ethical standards, both legislative and nonlegislative, must be complied with if applicable, such as privacy/personal data protection laws (e.g., General Data Protection Regulation in the EU, and Health Insurance Portability and Accountability Act, Health Information Technology for Economic and Clinical Health Act, and 21 CFR Part 11 in the United States), patent laws, ownership laws, UNESCO Declaration on the Human Genome and Human Rights, OECD Guidelines on Human Biobanks and Genetic Research Databases, and relevant local ethics regulations and policy measures on the protection of human subjects in research (e.g., World Medical Association Declaration of Helsinki and Declaration of Taipei, the Ethical Guidelines for Medical and Health Research Involving Human Subjects in Japan, and Common Rule in the United States).

## A Proposed Model Ethical Policy

Based on the considerations above and thorough institutional discussions, we have formulated and made public an ethics policy for the NCC J-PDX Library (Table 1). We believe that it is a social responsibility for an individual PDX repository to properly prepare its own code of ethics or institutional ethics policy on PDX models in promoting ethical implementation and utilization of these models.

**Table 1.** Model Ethical Governance Policy for the NCC J-PDX Library.

The Ethics Policy on the Implementation and Use of the J-PDX Library
Preamble
The usefulness of patient-derived xenograft (PDX) models, particularly those with individual patients’ detailed clinical information, for drug discovery and development as well as for medical research, has recently garnered attention domestically and internationally. On one hand, PDX samples can be proliferated by transplantation or passaging into experimental animals, enabling repeated use of the samples over a long period of time. On the other hand, these characteristics mean that PDX models always carry the risk of being provided to unspecified irresponsible third parties without controls or limits, and they inevitably carry a potential concern of abuse, thus impairing the patient’s dignity. In light of a recent series of scandals involving leaks and disclosures of personal information, there are strong calls by society to properly manage and protect privacy and personal information. In response to this social trend, the Act on Protection of Personal Information has been repeatedly reinforced through amendments, and penalties for infringement of personal information are becoming tougher each year. Moreover, researchers have now widely acknowledged that biomedical research involving human biospecimens and clinical information, as well as long-term research infrastructure such as biobanks that collect, store, provide, or distribute those specimens, and that information for other research projects and drug development purposes must establish an ethical research framework based on trust and social acceptance. Considering these circumstances, the National Cancer Center Japan (NCC) has established *the Ethics Policy*. In compliance with this policy, the NCC will ensure the promotion of the development and use of PDX models that are not just convenient but also scientific, easy to use, and ethically appropriate.
Policies
1. Safeguarding the dignity of individual patients in relation to PDX models
PDX models and clinical information stored in the J-PDX Library are derived from individual patients who are to be treated with dignity. Thus, these samples and that information should not be treated as mere objects or data but as “human-derived” materials in compliance with the Ethical Guidelines for Medical and Health Research Involving Human Subjects (MEXT, MHLW, and METI Notice no. 1 of 2021). The NCC and users of the Library must always be aware that their greatest responsibility is safeguarding the dignity of individual patients who contribute to the Library.
2. Trust-based management and governance of PDX models
PDX models and clinical information stored in the J-PDX Library are a valuable resource for medical research and development of pharmaceutical products, and they are entrusted to the NCC by individuals who agree with the mission and purpose of the Library. Thus, the NCC will be responsible for operating the Library ethically and providing them only to trustworthy users, including academia and the medical industry, which will use them for the public good in accordance with the goals of the Library.
3. Temporary lending of PDX models for limited purposes
The provision of PDX models and clinical information in the J-PDX Library to Library-users will be limited to temporary lending for limited purposes of proper use based on a contract between the users and the NCC to prevent unethical behavior by users such as the sale or subleasing of human-derived materials. The NCC will properly monitor and manage the use of PDX models by users with utmost attention.
4. Ethical considerations in the care and use of laboratory animals
Laboratory animals are essential for the construction and implementation of the J-PDX Library. The NCC will take care of the welfare of laboratory animals used for PDX models, refining how PDX models are created to minimize potential pain and distress for the animals, using methods to minimize the number of animals used, and making efforts to find and use better alternatives when possible. Everyone involved in the Library must never forget to be grateful for the animals that make the Library possible.
5. Compliance with relevant laws, ordinances, and regulations
In the implementation of J-PDX Library, the NCC will comply with relevant national and international laws, ordinances, and regulations, such as those concerning personal data protection, protection of human subjects, laboratory animal welfare, and intellectual property.

## Conclusion

In this study, we discussed the importance of establishing an ethical policy for the implementation and use of human-in-mice PDX models, providing a model ethical policy established for the NCC J-PDX Library. We hope that the ethical handling of PDX models will be promoted in accordance with the continuous growth of social expectations for research using PDX models.

## Article Information

### Conflicts of Interest

None

### Sources of Funding

This work was supported by AMED under Grant Number JP 23mk0101225.

### Acknowledgement

 We thank the members of the NCC J-PDX Library Steering Committee for their helpful discussions on ethics for the PDX models in the draft policy development process.

### Author Contributions

Conception of the work: AH, SY, and KM

Draft writing of the presenting policy in Figure 1: KM, SY

Writing of the paper: KM

Critical revision of the paper: SY, and AH

All authors have read the final draft and approved it for submission.

### Approval by Institutional Review Board (IRB)

Not applicable
